# Selection of suitable housekeeping genes for expression analysis in glioblastoma using quantitative RT-PCR

**DOI:** 10.1186/1471-2199-10-17

**Published:** 2009-03-03

**Authors:** Valeria Valente, Silvia A Teixeira, Luciano Neder, Oswaldo K Okamoto, Sueli M Oba-Shinjo, Suely KN Marie, Carlos A Scrideli, Maria L Paçó-Larson, Carlos G Carlotti

**Affiliations:** 1 Department of Surgery and Anatomy, Faculty of Medicine, University of São Paulo, Av. dos Bandeirantes 3900, 140490-900, Ribeirão Preto, SP, Brazil; 2 Department of Cellular and Molecular Biology, Faculty of Medicine, University of São Paulo, Av. dos Bandeirantes 3900, 140490-900, Ribeirão Preto, SP, Brazil; 3 Department of Pathology, Faculty of Medicine, University of São Paulo, Av. dos Bandeirantes 3900, 140490-900, Ribeirão Preto, SP, Brazil; 4 Department of Neurology and Neurosurgery, Federal University of São Paulo, R. Botucatu 740, 04023-900, São Paulo, SP, Brazil; 5 Department of Neurology, School of Medicine, University of São Paulo, Av. Dr. Arnaldo 455, 01246903, São Paulo, SP, Brazil; 6 Department of Pediatrics, Faculty of Medicine, University of São Paulo, Av. dos Bandeirantes 3900, 140490-900, Ribeirão Preto, SP, Brazil

## Abstract

**Background:**

Considering the broad variation in the expression of housekeeping genes among tissues and experimental situations, studies using quantitative RT-PCR require strict definition of adequate endogenous controls. For glioblastoma, the most common type of tumor in the central nervous system, there was no previous report regarding this issue.

**Results:**

Here we show that amongst seven frequently used housekeeping genes TBP and HPRT1 are adequate references for glioblastoma gene expression analysis. Evaluation of the expression levels of 12 target genes utilizing different endogenous controls revealed that the normalization method applied might introduce errors in the estimation of relative quantities. Genes presenting expression levels which do not significantly differ between tumor and normal tissues can be considered either increased or decreased if unsuitable reference genes are applied. Most importantly, genes showing significant differences in expression levels between tumor and normal tissues can be missed. We also demonstrated that the Holliday Junction Recognizing Protein, a novel DNA repair protein over expressed in lung cancer, is extremely over-expressed in glioblastoma, with a median change of about 134 fold.

**Conclusion:**

Altogether, our data show the relevance of previous validation of candidate control genes for each experimental model and indicate TBP plus HPRT1 as suitable references for studies on glioblastoma gene expression.

## Background

Methods for the quantification of accurate gene expression have an increasingly important role in studies aiming for the reliable examination of expression profiles generated by high-throughput approaches. Real-time reverse transcription quantitative PCR (qRT-PCR) has emerged as one of the most powerful tools for this purpose. Given the extreme sensitivity of qRT-PCR, a careful and stringent selection of a proper constitutively expressed control gene is required to account for differences in the amount and quality of starting RNA and in cDNA synthesis efficiency. Adequate normalizations presume the use of an internal control, often referred to as a housekeeping or reference gene, whose expression levels should not significantly vary among tissues and experimental situations analyzed [1,2]. Genes most commonly applied as references in qRT-PCR studies include: beta actin (*ACTB*), glyceraldeyde-3-phosphate dehydrogenase (*GAPDH*), beta glucuronidase (*GUSB*), hypoxanthine guanine phosphoribosyl transferase (*HPRT1*) and ribosome small subunit (*18S*) ribosomal RNA [1-3]. However, several reports have mentioned these classical housekeeping genes as showing variable expression levels in different experimental conditions [3-9]. Furthermore, the same gene revealed as almost invariant for certain tissues or cell types or could present highly variable expression levels in other tissues or experimental conditions [2,9,10]. Thus, it is clear that suitable control genes are extremely specific for particular sample sets and experimental models, being a crucial component in assessing confident gene expression patterns. It has been strongly suggested that more than one stable expressed reference gene should be used to avoid misinterpretation of gene expression data [6,7,11-13].

In this context, the present work aimed to evaluate suitability of selected candidate housekeeping genes for expression analysis in glioblastoma (GBM), the highest-grade malignant astrocytoma [14]. These malignant gliomas are the most common and the major lethal type of tumor in the central nervous system [15], leading to a mean survival time of 1 year after diagnosis [16]. This discouraging prognosis is decurrent from both the infiltrative nature of the tumor and the resistance of tumor cells to cytotoxic treatments [17-19]. Many therapy modalities based on characterized genetic alterations are already in use or in clinical trials phase, but their efficacy is still below expectation [19-21]. Thus, the need for novel therapeutic targets for GBM treatment becomes urgent. In this direction, several recent studies are dedicated to explore high-throughput expression profiles, using qRT-PCR to produce reliable measurements, in order to identify novel genes differentially expressed in GBM [22-26].

Although the necessity of stringent selection of housekeeping genes is well established, until now it has been no systematic investigation directed to point out adequate control genes for quantitative expression analysis in GBM. The majority of the studies apply one of the most commonly used housekeeping genes, such as *ACTB *and *GAPDH*. To get the actual panorama of reference genes used in GBM quantitative expression studies, we performed a Medline search using the terms real-time PCR and glioma. We found 45 available articles, published from January 2007 to July 2008, based on the use of different reference genes. More than 80% of these studies use one of the following genes as internal controls: *ACTB *(cited 19 times, 42%), *GADPH *(cited 13 times, 29%) or *18S rRNA *(cited 5 times, 11%), without any previous evaluation of their stability within the model. This search revealed that we do not have a consensus in the field and, moreover, a meaningful study on the application of reference genes in glioblastoma gene expression investigation is essential and timely.

Therefore, we investigated here the suitability of seven frequently used housekeeping genes for real-time RT-PCR analysis in human GBM *versus *non-neoplastic white matter comparisons. We determined that TBP and HPRT1 are suitable reference genes for expression studies in GBM. The significance of applying adequate normalization methods was demonstrated by the evaluation of the expression levels of 12 target genes upon different normalization approaches. Our data revealed that, depending on the normalization method utilized, genes whose expression levels are similar in normal and tumor tissues could be interpreted as up or down regulated and genes presenting significant differences in expression levels can be missed. These data show the relevance of previous validation of candidate control genes to obtain adequate normalizations in quantitative expression studies.

## Results

### The Expression Levels of Candidate Housekeeping Genes

**Figure 1:**
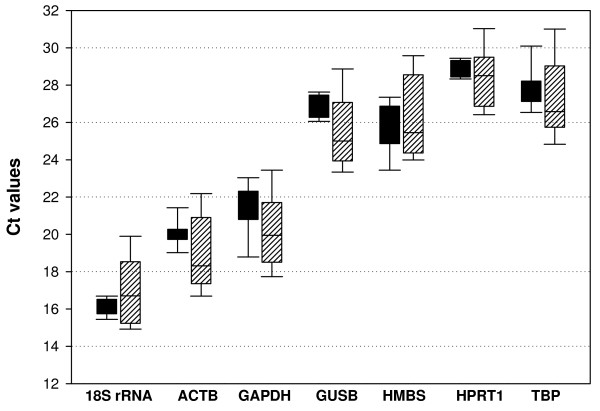
**Expression levels of candidate housekeeping genes in glioblastoma and non-neoplastic white matter**. Boxes represent lower and upper quartiles of cycle thresholds range with medians indicated, whiskers represent the 10^th ^and 90^th ^percentiles. Black boxes correspond to non-neoplastic white matter samples and hatched boxes to tumor samples. Graph was plotted with Sigma Plot 10.0 software.

We chose to investigate seven housekeeping genes commonly used as internal controls in expression studies, *ACTB*, *GAPDH*, *GUSB*, *HMBS*, *HPRT1*, *TBP *and *18S rRNA *(Table 1). According to articles published over the past two years, three of them, ACTB, GAPDH and 18S rRNA, collectively correspond to the endogenous controls applied in more than 80% of expression analyses performed on glioma tumors or cell lines. Transcriptional levels of the seven selected genes were determined in a panel of 39 microdissected samples from different individuals, nine non-neoplastic white matter and 30 glioblastomas, using real-time RT-PCR. For comparison of housekeeping transcription levels, the cycle threshold (C_T_) values were plotted directly, assuming the same threshold for all genes evaluated. The C_T _is defined as the number of cycles needed for fluorescence to reach a specific threshold level of detection and is inversely correlated with the amount of RNA template present in the reaction. The seven housekeeping genes analyzed here displayed a wide expression range, with C_T _values between 14 and 32 (figure 1). The C_T _values for all these genes showed normal distribution according to the Kolmogorov and Smirnov method, in both tumor and non-neoplastic samples. These genes are clearly distributed into different expression level categories. The extremely abundant *18S rRNA*, which represents the bulk of total RNA in the cell, presented C_T _values below 18 cycles; genes coding for highly expressed mRNAs, such as *ACTB *and *GAPDH*, with majority of C_T _values between 18 and 22 cycles; and the moderately expressed genes, *GUSB*, *HMBS*, *HPRT1 *and *TBP*, showing C_T _values between 26 and 30 cycles. We also noted slightly higher levels in the abundance of *GUSB *and *HPRT1 *mRNAs than in *HMBS *and *TBP *mRNAs. For all candidate control genes analyzed, the amplitudes in expression ranges were of about 3–4 cycles larger in GBM than in non-neoplastic samples (figure 1), pointing out the great variability in gene expression levels peculiar of heterogeneous cancer tissues even for those so called housekeeping genes.

**Table 1:** Selected housekeeping genes for expression analysis

**Gene name**	**Gene symbol**	**Accession number**	**Function**
Beta-actin	*ACTB*	NM_001101	Cytoskeletal structural protein
Glyceraldeyde-3-phosphate dehydrogenase	*GAPDH*	NM_002046	Glycolysis enzyme
Beta-glucuronidase	*GUSB*	NM_000181	Exoglycosidase in lysosomes
Hydroxymethylbilane synthase	*HMBS*	NM_000190	Heme biosynthetic pathway
Hypoxanthine guanine phosphoribosyl transferase 1	*HPRT1*	NM_000194	Metabolic salvage of purines
TATA-box binding protein	*TBP*	NM_003194	General transcription factor
18S ribosomal RNA	*rRNA*	NR_003286	Ribosome subunit

### Stability of Candidate Housekeeping Genes on Normal and Neoplastic Brain Tissues

Our main objective was to identify housekeeping genes with minimal variability among our set of samples, which includes non-neoplastic white matter and glioblastoma tumors. In order to determine the least variable reference genes, we evaluated expression stability of the seven candidate controls in our panel of samples, by the geNorm software analysis. GeNorm calculates a gene-stability measure (M) based on the average pairwise variation between a particular gene and all other genes studied. High expression stability is indicated by a low M value as an estimate of combined variation of the individual gene. Successive elimination of the least stable gene ranks the candidate housekeeping genes according to their M values and identifies the two most stable reference genes [13]. The M values calculated by geNorm for the seven candidate endogenous controls are shown in table 2. All analyzed genes reached M values below the default limit of 1.5 suggested in the geNorm program. After stepwise exclusion of the least stable genes from bottom to top, *ACTB *and *GUSB *were found to be the two most stable reference genes, with paired M equal to 0.56, followed by *TBP *with M equal to 0.736.

**Table 2:** Expression stability measures (M) calculated by geNorm for all candidate housekeeping genes analyzed

**Ranking order**	**Gene**	**M values^1^**
1	*ACTB*	0.603
2	*GUSB*	0.693
3	*TBP*	0.736
4	*GAPDH*	0.918
5	*HPRT1*	1.049
6	*18S rRNA*	1.277
7	*HMBS*	1.344
Best combination of two genes	*ACTB *+ *GUSB*	0.560

^1 ^Lower M values indicate higher expression stability

**Figure 2:**
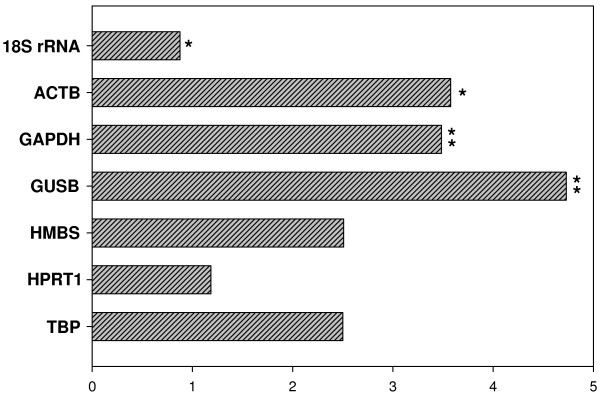
**Expression levels fold changes of candidate housekeeping genes in tumor *versus *normal tissues**. Bars show the ratios of median expression levels between tumor and normal tissues for the indicated housekeeping genes. Asterisks indicate the significance of differences, * P values < 0.05 and ** P values < 0,005. Graph was plotted with Sigma Plot 10.0 software.

However, if we compared raw C_T _values of tumor versus non-neoplastic samples, as suggested by Ohl F. and collaborators (2005), significant differences in gene expression between GBM and normal white matter were found for *ACTB *(Student's t test, P = 0,016), *GAPDH *(P = 0,006), *GUSB *(P = 0,005) and *18S rRNA *(P = 0.012). *ACTB*, *GAPDH *and *GUSB *mRNA levels are significantly increased in tumor samples, with changes of about 3.6, 3.5 and 4.7 fold, respectively; while *18S rRNA *was revealed to be slightly diminished in tumor samples, in a proportion of approximately 13% (figure 2). The encountered differences reveal that *ACTB*, *GAPDH*, *GUSB *and *18S rRNA *are inadequate control genes for normalization purposes in profiling studies comparing GBM to the normal counterpart. These four genes were consequently excluded from geNorm analysis and the three candidate controls whose expression levels did not significantly vary between normal and tumor tissues (*HMBS*, *HPRT1 *and *TBP*) were reevaluated by the geNorm software. The M values calculated by geNorm for these three endogenous control candidates are 1.423, 1.247 and 1.047 for *HMBS*, *HPRT1 *and *TBP*, respectively. Thus, *TBP *and *HPRT1 *were indicated by geNorm as the two most suitable reference genes, presenting a combined M value of 0.871, much lower than the 1.5 suggested cut off (table 3).

**Table 3:** Expression stability values calculated by geNorm and NormFinder for the three genes expressed in similar levels between tumor and normal tissues

**Gene**	**geNorm**	**NormFinder**
*HMBS*	1.344	0.298
*HPRT1*	1.049	0.356
*TBP*	0.736	0.164
Best combination of two genes	*TBP *+ *HPRT1 *= 0.87	*TBP *+ *HPRT1 *= 0.166

**Figure 3:**
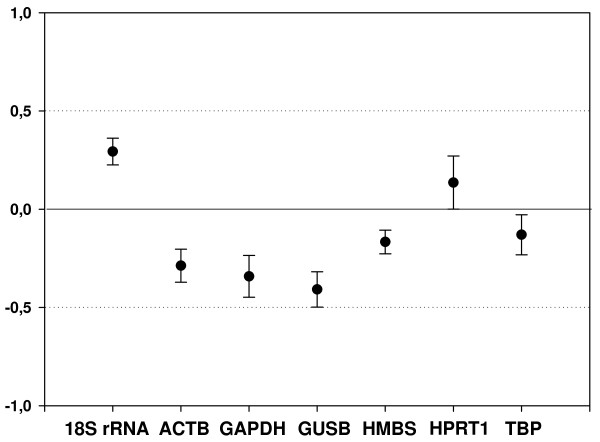
**Equivalence test for the seven candidate housekeeping genes in the white matter and GBM sample groups**. The differences of means (solid circles) and the matching symmetrical confidence intervals (whiskers) are shown for the logarithmized relative expression of each reference gene. Y-axis represents the fold changes in expression levels between tumor and normal tissues. The deviation area from -1 to 1 indicates fold changes ≤ 2. If the symmetrical confidence interval is included in the deviation area and contains zero, the gene is considered equivalently expressed.

Expression stability of *HMBS*, *HPRT1 *and *TBP *genes were additionally evaluated with NormFinder, other software that uses a model-based approach to measure gene expression variation among sample subgroups [27]. NormFinder calculates stability values for each analyzed gene on the basis of inter- and intragroup expression variation. The lower stability values indicate the more stable expressed candidate genes. Although this analysis revealed that TBP and HMBS show the lower isolated stability values, NormFinder also indicates *TBP *and *HPRT1 *as the best combination of two genes for normalizing calculations, with a combined gene stability value of 0.166 (table 3). We also performed the equivalence test [28] to estimate the significance of differences in the median expression values between tumor and normal tissues of each individual gene. We observed that among all genes studied *TBP *and *HPRT1 *were confirmed as the more equivalently expressed, once the confidence intervals they presented are included in deviation area and are closest to zero (figure 3). Therefore, it can be concluded that normalization using these two reference genes is an adequate approach for gene expression studies in GBM.

### Evaluation of the Expression Profiles of Target Genes Following Different Normalization Approaches

**Figure 4:**
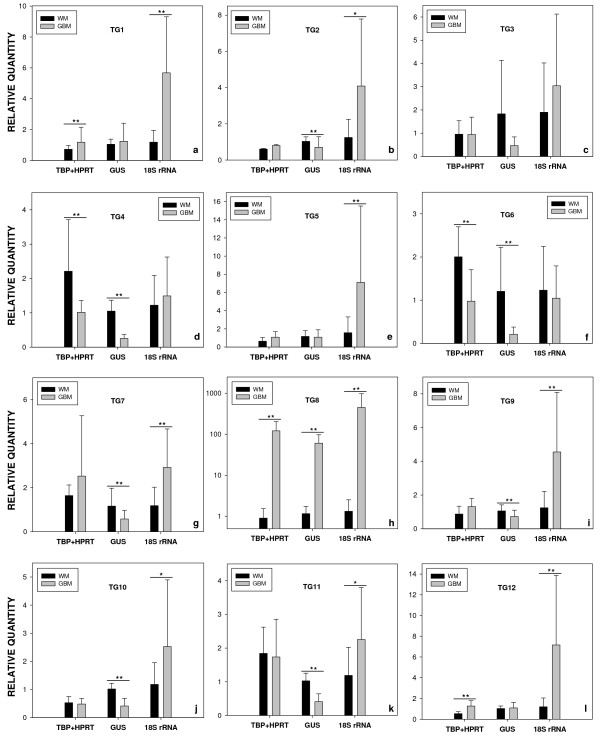
**Expression levels of target genes in normal and tumor tissues upon different normalization approaches**. Median relative quantities of target genes (TG1–12) in non-neoplastic white matter (black bars) and glioblastoma (gray bars) samples were plotted after normalization under the indicated conditions: with geNorm normalization factors calculated from *TBP *plus *HPRT1 *and with the genes *GUSB *or *18S rRNA *alone. Whiskers indicate the standard deviation. Significance between differences was calculated by the use of Mann-Whitney test. Asterisks indicate the significance of differences, * P values < 0.05 and ** P values < 0,005. Graphs were plotted with Sigma Plot 10.0 software.

We have performed different normalization approaches to a set of target genes to demonstrate the importance of using suitable housekeeping genes in order to get the correct expression profiles. Based on data from a previously analyzed microarray [26], we selected 12 target genes, candidates of being over-expressed in GBM, for quantitative RT-PCR experiments to investigate reliability of over-expression suggestion. According to microarray data, these 12 selected genes presented changes of tumor *versus *normal expression levels higher than 25 fold and, at the beginning of this study they had no molecular characterization, being classified as unknown function after Gene Ontology analysis. Thus, this set of genes represented putative novel genes involved in GBM development. Table 4 gives identification of the 12 analyzed target genes and summarizes their current annotation status [29-38]. Expression levels of these target genes were determined in our panel of glioblastoma and non-neoplastic white matter samples. Normalization was performed using five different methods: with normalization factors calculated by geNorm considering *TBP *plus *HPRT1 *as references, with *TBP *and *HPRT1 *separately, and with the two unstable genes, *GUSB *and *18S rRNA*, which are 4.7 fold increased and 13% decreased, respectively, in GBM when compared to normal white matter. In general, expression levels of target genes in GBM and normal white matter when using *TBP *plus *HPRT1*, or either *TBP *or *HPRT1 *alone show similar pattern, but in several cases the significance of differences vary (see additional file 1), demonstrating that the simultaneous use of two adequate reference genes is indicated. If we compare the expression profiles obtained with the most confident normalization approach (*TBP*+*HPRT1*) to the expression profiles given by using either *GUSB *or *18S RNA*, we will observe dramatic differences (figure 4). Among seven genes whose expression levels did not significantly vary between tumor and normal tissues, five (71%) would be considered significantly diminished when normalized with *GUSB *(figure 4B, G, I, J and 4K) and six (86%) would be considered significantly increased when normalized with *18S rRNA *(figure 4B, E, G, I, J and 4K). Additionally, the two genes that indeed showed higher quantities in GBM could not be detected when normalized with *GUSB *or could have an overestimated increase when normalized with *18S rRNA *(figure 4A and 4L). Similar misinterpretation could occur in the case of the two genes presenting lower quantities in GBM, which would be considered as not significantly differing or could have an overestimated decrease, when normalized with either *18S rRNA *or *GUSB*, respectively (figure 4D and 4F). The normalization method applied did not significantly alter only the expression profile of NM_018410 (TG8), whose mRNA quantities are enormously higher in tumor than in normal tissue (figure 4H, note that the graph is presented in logarithmic scale). Despite that, even in this case, we observed different ratios of expression level when normalizing with *TBP*+*HPRT1 *(134 fold), *GUSB *(52 fold) or *18S rRNA *(340 fold). As our data has shown, among 12 genes analyzed, 10 (83%) presented different expression profiles depending on the normalization approach utilized. These data reveal that the use of inadequate endogenous control could have a significant impact on the evaluation of target gene expression levels, in many cases giving contrary results, especially for those presenting small differences between tumor and normal tissue.

**Table 4:** Target genes evaluated in expression analysis

**Code**	**Accession number**	**Gene symbol**	**Functional/domain information**	**Reference**
TG1	NM_001080522	*CC2D2A*	coiled-coil and C2 domain containing 2A	Noor A et al., 2008 [29]
TG2	NM_017925	*DENND4C*	DENN/MADD domain containing 4C	Olsen JV et al., 2006 [30]
TG3	NM_024759	*NPAL2*	NIPA-like domain containing 2	Lefrève, C et al., 2004 [31]
TG4	NM_022831	*AIDA*	axin interactor, dorsalization associated	Rui Y et al., 2007 [32]
TG5	NM_024857	*ATAD5*	ATPase family, AAA domain containing 5	Douglas, J et al., 2007 [33]
TG6	NM_024859	*MAGIX*	MAGI family member, X-linked transcript variant 1	Ota T et al., 2004 [34]
TG7	NM_018093	*WDR74*	WD repeat domain 74	Eilbracht J et al., 2004 [35]
TG8	NM_018410	*HJURP*	Holliday junction recognition protein	Kato T et al., 2007 [36]
TG9	NM_152622	*MIER3*	mesoderm induction early response 1, family member 3	Mehrle A et al., 2006 [37]
TG10	NM_024942	*C10 or f88*	chromosome 10 open reading frame 88	Gerhard, DS et al., 2004 [38]
TG11	NM_138341	*TMEM116*	transmembrane protein 116	Gerhard, DS et al., 2004 [38]
TG12	NM_018087	*TMEM48*	transmembrane protein 48	Olsen JV et al., 2006 [30]

## Discussion

The present study is the first report of a systematic evaluation of potential reference genes with regard to their usefulness as normalizers in malignant glioma expression studies. Amongst seven commonly used classical housekeeping genes, we found that expression levels of *ACTB*, *GAPDH*, *GUSB *and *18S rRNA*, significantly differed between tumor and normal tissues on the basis of the examination of raw C_T _values (figure 2). It could be concluded that these genes are regulated and thus not indicated for target gene normalizations. It was previously reported that this initial analysis is mandatory in order to exclude highly unstable genes from further calculations using programs based on pairwise variation, such as geNorm and NormFinder [7,39]. In those studies, Ohl and collaborators have found genes up and down regulated in prostate and bladder cancer samples through comparisons of C_T _values. We also consider this preliminary exclusion crucial, because the simultaneous analysis of several genes whose expression levels are similarly biased, could lead to the wrong choice on the basis of software evaluation only. This type of misinterpretation can be clearly illustrated by data presented in table 2, where *ACTB *and *GUSB *are being indicated as the two best reference genes. *ACTB *and *GUSB *selection, based on geNorm analysis including the seven potential housekeeping genes, probably resulted from similarities in the expression pattern of these two genes, which are both significantly up regulated in tumor tissues, as well as *GAPDH *(figure 2). Although such genes that regulate basic and ubiquitous cellular functions are frequently assumed as almost invariable between different samples, many other studies corroborate our observations and have also demonstrated that their individual expression may vary as a result of neoplastic growth, hypoxia or experimental treatment [1,3,5,6,11]. These data show the obligatory requirement of prior exclusion of regulated genes based on raw expression data evaluation.

Among the seven candidate housekeeping genes analyzed, *TBP *and *HPRT1 *were indicated as the best combination of reference genes for expression studies in GBM, using three independent methods of analysis: geNorm [13], NormFinder [27] and equivalence test [28]. Recently, both *HPRT1 *and *TBP *were indicated as suitable reference genes for differential expression studies using qRT-PCR in different type of cancers, moreover *HPRT1 *was recommended as a universal single reference gene for expression analysis in cancer [3,7,39]. However, normalizations based in more than one best-performing reference gene gives more accurate results and has been increasingly suggested [6,13,40]. In our study, we observed differences in the significance of comparisons when utilizing *TBP *and *HPRT1 *associated or each one separately (see additional file 1). Thus, until further extended analysis becomes available, we suggest the use of *TBP *plus *HPRT1 *as the more adequate endogenous controls for target gene normalizations in GBM expression analysis.

The significance of applying different reference genes for the estimation of the relative quantities of gene expression, was demonstrated here by the analysis of 12 target genes, candidates of being over expressed in GBM, following three normalization approaches: i) with geNorm normalization factors calculated for *TBP*+*HPRT1*; ii) with *GUSB*, which is ~4.7 times increased in tumors; and iii) with *18S rRNA*, which is ~13% decreased in GBM samples. Our data clearly show that the normalization method applied might introduce errors in the estimation of relative expression levels. Genes, whose expression levels did not significantly vary between tumor and normal tissues, would be considered significantly diminished when normalized with *GUSB *or increased when normalized with *18S rRNA*. Moreover, genes presenting significant differences in the relative quantities between tumor and normal tissues can be missed if these unsuitable endogenous controls are utilized (figure 4). Therefore, we can conclude that for GBM gene expression studies, *GUSB *along with the most frequently utilized internal controls, *ACTB *and *GAPDH*, must be considered inadequate for normalizations due to its significant increase in tumor samples. *18S rRNA *also led to erroneous estimation in gene expression levels and proved not to be useful for normalizations. This could be explained by the imbalance between messenger and ribosomal RNA [41] or, possibly, by the independently regulated rRNA transcription, which is carried out by RNA polymerase I, as previously reported [1].

In this study, we found five genes whose expression levels significantly differ between tumor and normal samples. Surprisingly, two of them were decreased (TG4 and TG6) and three (TG1, TG8 and TG12) were increased in tumor tissue, and only one confirmed the high expression levels (>25 fold) indicated by the microarray data [26]. This is probably due to the difference in the sizes of the tumor samples analyzed that was five times smaller in the microarray experiments (n = 6) than in the qRT-PCR analysis performed here (n = 30). We also observed that the validation rate of the microarray data obtained in our study (25%) was much lower than previously reported (90%) [26]. The high validation rate reported in the former study can be explained by the criteria of target genes selection, which was biased to genes related to pathways probably altered in cancer. One of the three genes validated here, NM_018410, presents transcription levels extremely elevated in GBM, independently of the reference gene utilized (TG8, figure 3H). Over-expression of TG8 in GBM when compared to pilocytic astrocytoma was previously suggested by microarray data analysis, where a change of about nine fold was observed [42]. This gene was recently annotated as *HJURP*, the *Holliday Junction Recognition Protein*. It was demonstrated that HJURP is over expressed in lung cancer and is involved in chromosomal stability, being a competence factor for immortality of cancer cells in culture [36]. The role of HJURP in glioblastoma will be further characterized.

## Conclusion

In conclusion, our data show the relevance of previous validation of candidate housekeeping genes for each specific application, especially when small differences are intended to be detected. For glioblastoma, it was demonstrated that *TBP *plus *HPRT1 *are suitable reference genes for normalization purposes in gene expression profiling studies. Together, these results highlight the importance of careful reevaluation of glioblastoma gene expression data currently available.

## Methods

### Tissue samples

Glioblastoma samples were obtained from 30 patients (mean age 55 years, range 19–79 years) submitted to surgical resection for tumor ablation at the Clinical Hospital of the Faculty of Medicine of Ribeirão Preto, University of São Paulo. Tumor grade was determined according to WHO criteria [14]. Non-neoplastic white matter samples were obtained from patients undergoing temporal lobectomy for epilepsy treatment. The study was approved by the Ethics Committee of the Faculty of Medicine and informed consent was obtained from each patient. Thirty tumors and nine non-neoplastic fresh surgical samples were sectioned and immediately snap-frozen in liquid nitrogen upon surgical removal. All tissue samples were microdissected for exclusion of tissue areas presenting necrosis or not matching to GBM diagnostic prior to RNA extraction. Standardized conditions of storage and microdissection of tumor samples are important steps to guarantee reliability of data and the conclusions derived from them, since GBM are heterogeneous solid tumors often presenting necrosis [16].

### RNA Isolation and Quality Evaluation

Total RNA was isolated using the TRIzol Reagent (Invitrogen) following the manufacturer's instructions with an additional phenol/clorophorm extraction to improve protein exclusion. The concentration and purity of isolated RNA were assessed by absorbance (A) readings on a UV spectrophotometer (Hitachi) at the wavelengths of 260 and 280 nm. The mean ratio value of A_260/280 _for all RNA samples was 1.81 (± 0.06), reflecting high purity and protein absence. RNA integrity was evaluated by the ratio of 28S/18S ribosomal RNA bands after eletrophoresis in denaturing 1% agarose gel. To guarantee the quality necessary for expression analysis all samples used in this study presented a 28S/18S rRNA ratio ≥1.7.

### DNAse Treatment and cDNA synthesis

One microgram of total RNA from each sample was treated with DNAse I enzyme (Invitrogen) in the presence of 40 U of RNAse inhibitor (RNAseOUT, Invitrogen), following the instructions of the manufacturer. Treated RNA was reverse transcribed using the HighCapacity kit (Applied Biosystems) in 20 μL of final volume, according to fabricant's recommendations and with addition of 250 ng of oligo(dT)_18–24 _per reaction.

### Quantitative Real-Time RT-PCR

All primers were designed with OligoExplorer 1.2 software to amplify at 60°C and to bind specifically to different exons of human cDNA sequences. To evaluate the possibility of genomic amplification, minus-RT PCR were performed using DNAse treated RNA in the same dilution used for the cDNA samples. No amplification of the expected products were detected, except for the genes *18S rRNA*, which does not have introns, and *ACTB*, that presents pseudogene in the genome. However, the relative quantities obtained in minus-RT reactions were at least three orders of magnitude lower than in qRT-PCR performed with cDNA samples. Moreover, the amplification products were detected in similar levels in both GBM and normal white matter (control) samples. Primer sequences, the GenBank Accession numbers of target cDNAs, as well as the amplification reaction information are shown in table 5. The relative mRNA expression levels of target genes and candidate housekeeping genes were quantified using real-time PCR analysis with a Gene Amp^® ^7500 Sequence Detection System (PE Applied Biosystems).

**Table 5:** Primer sequences and amplification summary

**Gene**	**Primer Sequence [5' → 3']**	**Amplicon size (bp)**	**Intervening sequence size (bp)**	**Amplification efficiency (%)**
*ACTB*	F: GGCACCCAGCACAATGAAG R: CCGATCCACACGGAGTACTTG	66	178	98
*GAPDH*	F: AGATCCCTCCAAAATCAAGTGG R: GGCAGAGATGATGACCCTTTT	130	220	98
*GUSB*	F: GAAAATATGTGGTTGGAGAGCTCATT R: CCGAGTGAAGATCCCCTTTTTA	101	3360	93
*HMBS*	F: CACGATCCCGAGACTCTGCT R: TACTGGCACACTGCAGCCTC	81	315	104
*HPRT1*	F: TGAGGATTTGGAAAGGGTGT R: GAGCACACAGAGGGCTACAA	118	1833	99
*TBP*	F: GAGCTGTGATGTGAAGTTTCC R: TCTGGGTTTGATCATTCTGTAG	117	1747	110
*18S rRNA*	F: GGAGTATGGTTGCAAAGCTGA R: ATCTGTCAATCCTGTCCGTGT	129	_	89
TG1	F: AAGGTCGGAAGAAGGTGACAG R: GCTGCTGGAATTTGCTCACTG	120	4217	97
TG2	F: CTTTACCCAGCGACCGTTTCA R: GGACTCAAGTAGGGCACAGAA	123	2206	96
TG3	F: TACTCTGATCGCTCCGTTAGG R: CCTGCAAATGCCAGTGTCGTA	120	29982	92
TG4	F: AAAGATGCTGGGCAGTGCATC R: CCACAGGAGTATCTTGCACAG	94	2754	90
TG5	F: GCCAACCCTTCGAAACATCTG R: AGCTGCCAAAGTATTCACAGTC	130	242	110
TG6	F: AGCGCTGTGGTCGTTTGGAG R: GACGAATAACCAGGTGGAGCT	132	231	101
TG7	F: TTGCCACAGGTGGGAAAGAGA R: CAGTCATTCCGCACGTTCTTG	94	256	99
TG8	F: GAAGGGATGTACGTGTGACTC R: CCATTCTCTGGGAGATGAAGC	131	2129	98
TG9	F: GCCGAAGCTTTGAACATGCAC R: CACACTCAGCAACTGTCCTAG	93	4817	110
TG10	F: CTCTCCTGCTCTAGGATCAAG R: ATTCCGCTGCTGACACCTAAC	124	3241	96
TG11	F: GAACAGTGGGCAGTGATTCAC R: TTGGTGTCCTGTGGCTTAGTC	125	1351	87
TG12	F: CGGATTTCAGGAAGCCTTGTG R: GCAGATGCTTGCACAGCATTC	131	4426	90

F: forward primer, R: reverse primer

Amplification of specific PCR products was detected using the SYBR Green PCR Master Mix (PE Applied Biosystems) according to the manufacturer's protocol. All primers employed were synthesized by MWG Biotech Inc or Invitrogen. Amplification efficiency of each primer pair was evaluated by the standard curve method using serial dilutions of pooled cDNA. All primer pairs utilized in this study presented amplification efficiency between 87–110% (table 5). Reactions without template were run in parallel for all plates to verify purity of measurements within each experiment. Each run was completed with a melting curve analysis to confirm the specificity of amplification and lack of primer dimers. The 2^-ΔΔCT ^equation [43] was applied to calculate the relative expression of tumor samples and non-neoplasic brain tissues. Mean C_T _of non-neoplastic brain tissues was used as the calibrator sample.

### Statistical data analysis

The normality test was performed by the Kolmogorov and Smirnov method and significance between differences in mean C_T _values was measured by unpaired test t, using GraphPad InStat software. The differences in gene expression levels were analyzed by the Mann-Withney test, using the SPSS 15.0 software. P values < 0.05 were considered statistically significant. For evaluation of expression stability of the candidate reference genes, we applied the softwares geNorm [13] and NormFinder [27], and the equivalence test [28], as previously described. The geNorm and NormFinder programs are Visual Basic application tools for Microsoft Excel available on internet upon request to developers. C_T _values were converted into raw relative quantities considering the PCR efficiency 2.

## Authors' contributions

VV designed the study, performed the experiments and data analysis and primarily drafted the manuscript, SAT helped in sample collection and RNA extraction, LN performed microdissection and pathologic diagnosis of all tumor samples utilized, OKO carried out microarray data analysis, SMOS, SKNM and CAS contributed to study design and revised the manuscript, MLPL and CGC conceived the study, contributed to data analysis and helped to draft the manuscript. All authors read and approved the final text.

## Supplementary Material

Additional file 1 Expression levels of target genes in normal and tumor tissues upon different normalization approaches. Median relative quantities of the indicated target genes in non-neoplastic white matter (black bars) and glioblastoma (gray bars) samples after normalization with: geNorm normalization factors calculated from *TBP *plus *HPRT1 *and with the genes *TBP *or *HPRT1 *alone. Asterisks indicate the significance of differences, * P values < 0.05 and ** P values < 0,005. Click here for file
