# Supplementary figures and images for: Selection of suitable housekeeping genes for expression analysis in glioblastoma using quantitative RT-PCR
Source: BMC Mol Biol. 2009 Mar 3;10:17. doi: 10.1186/1471-2199-10-17 (PMC2661085; doi:10.1186/1471-2199-10-17)

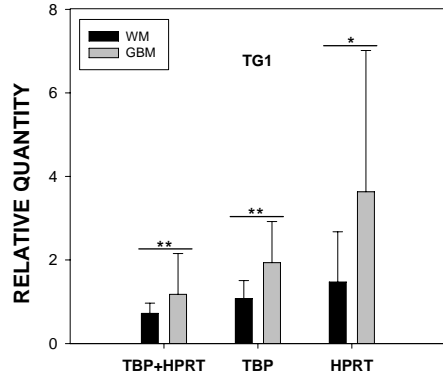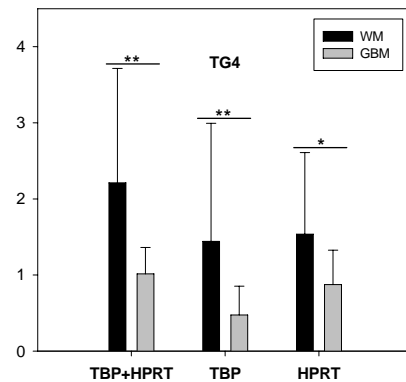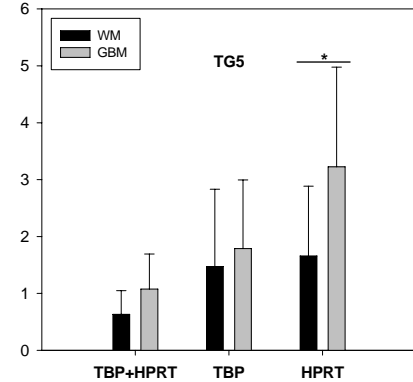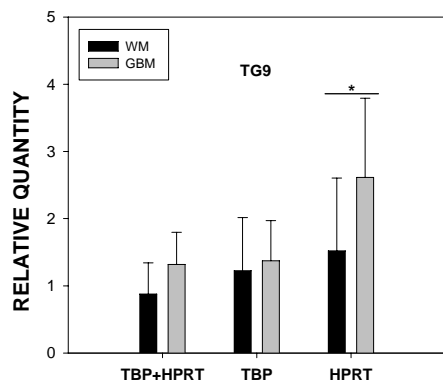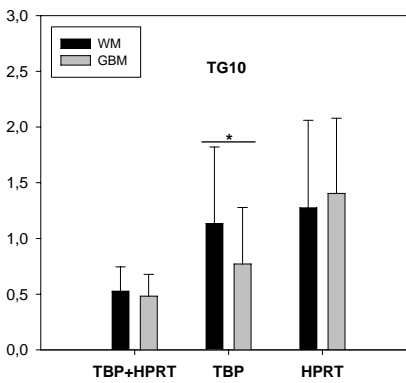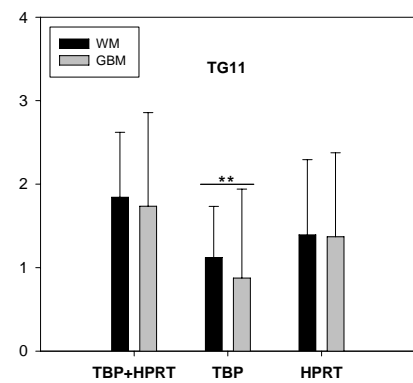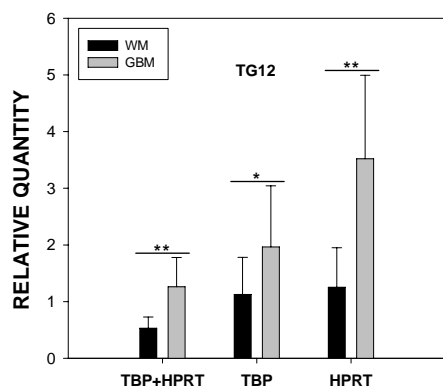

Supplement: Additional file 1 — Expression levels of target genes in normal and tumor tissues upon different normalization approaches. Median relative quantities of the indicated target genes in non-neoplastic white matter (black bars) and glioblastoma (gray bars) samples after normalization with: geNorm normalization factors calculated from TBP plus HPRT1 and with the genes TBP or HPRT1 alone. Asterisks indicate the significance of differences, * P values < 0.05 and ** P values < 0,005. [file 1471-2199-10-17-S1.pdf]
